# Synergistic tension among the music and lyrics of late-Romantic choral songs

**DOI:** 10.1038/s41598-026-50356-x

**Published:** 2026-05-29

**Authors:** Tudor Popescu, Adrian Kempf, Patrick Boenke, Marco Tettamanti

**Affiliations:** 1 Department of Cognition, Emotion, and Methods in Psychology, University of Vienna, Vienna, Austria; 2 Department of Psychology, University of Graz, Graz, Austria; 3 University of Music and Performing Arts, Vienna, Austria; 4 Department of Psychology, University of Milan-Bicocca, Milan, Italy; 5 Center for Mind/Brain Sciences (CIMeC), University of Trento, Rovereto, Italy

**Keywords:** Musical tension, Song lyrics, Narrative, Musical emotion, Prediction, Partial Information Decomposition (PID), Synergy, Cross-domain integration, Human behaviour, Cognitive neuroscience

## Abstract

Songs combine two defining human capacities: music and language. As a song unfolds, interconnected musical and linguistic elements, such as dissonant chords and evocative words, generate fluctuating patterns of tension and relaxation that shape listeners’ expectations and emotional experience. However, how these patterns integrate across the musical and linguistic domains to produce a unified sense of tension remains poorly understood. We investigated this question in a tension-rich subset of Western vocal music – late-Romantic choral songs – hypothesising that tonal and semantic tension combine superadditively. Using a naturalistic listening paradigm, online participants continuously rated tonal, semantic, and holistic tension while listening to three selected songs by Reger, Wolf, and Brahms. Holistic tension ratings were more closely predicted by tonal than by semantic tension. Information-theoretic analysis using Partial Information Decomposition further revealed significant *redundant* and *synergistic* informational contributions specifically in the Reger piece. This suggests that, under certain musical conditions – such as dense chromatic harmony combined with homophonic text setting – musical and linguistic cues can interact to produce integrated experiences of tension. Although based on a specific tonal-poetic idiom, these results further our understanding of how music and language may combine during real-time song listening from the experiential perspective of tension. We discuss how both culture-specific and potentially culture-invariant mechanisms may contribute to this cross-domain aesthetic integration.

**Supplementary Information:**

The online version contains supplementary material available at 10.1038/s41598-026-50356-x.

## INTRODUCTION

Music and language (**M&L**) are among our species’ most distinctive cognitive capacities. Both domains organise perceptually discrete elements into hierarchical structures, following syntactic principles^1^. The temporal unfolding of these syntactic sequences engages predictive processes that ultimately convey meaning, emotion, and aesthetic experience^2–4^. These structural and evolutionary affinities have inspired extensive theoretical work on the comparative study of M&L (e.g.^5–9^). A substantial empirical literature has compared behavioural (e.g.^10^) and neurophysiological (e.g.^11,12^) reactions to carefully controlled musical and linguistic stimuli, often attempting to specify purported shared cognitive and neuronal resources that reflect these affinities^13–15^.

Songs with lyrics represent particularly relevant objects of study in this context. While some neuropsychology studies suggest independent, dual encoding of lyrics and melody in songs^16,17^, more recent evidence points to their perceptual connectedness in memory^18,19^, underscoring the complexity of their integration during song listening. Songs offer an optimal naturalistic tool for studying the *affective* response to M&L specifically^20–22^. Akin to a narrative unfolding^23^, distinct features of M&L, such as chords of varying dissonance and words of varying affective valence, combine in songs to create patterns of tension-relaxation that drive a powerful *unified* affective experience. How M&L features integrate during song listening to engender affect remains however an open question. The interplay between expectation and its degree of fulfillment, thought to create our emotional response to song, reflects a feature of the predictive brain^24^, whose complexity is not fully understood.

In Western music, tension is a multifaceted construct driven by both music-structural features and psychoacoustic parameters. Tonal relations – the melodic and harmonic motion between pitch events – are major contributors to this experience, as movements away from a stable “home” chord or the use of unresolved dissonances can tense the music by violating listener expectations^25,26^. Additional contributors include non-tonal structural features, such as rhythm and meter, and interpretative elements like loudness, tempo, and texture^27–31^. Despite this complexity, musical tension holds intuitive appeal and demonstrates high reliability across listeners, bridging music-theoretical concepts with objective physiological measures of arousal^31–34^. The present study focuses primarily on music-structural dimensions of tension, particularly prominent in the late-Romantic repertoire used here. While participants rated musical tension holistically, our analysis seeks to isolate the contributions of compositional structure from low-level acoustic fluctuations. To this end, we treated non-structural variables, such as loudness, as nuisance factors by including the audio signal’s loudness envelope as a regressor in our models. This approach allows for a clearer estimation of how the interplay between compositional architecture and linguistic narrative generates perceived tension.

In songs, affective meaning compounds across the dimensions structuring musical and textual events. In text-setting practices that match words to music, linguistic units (syllables) typically align with musical units (pitch onsets). This generally coordinates stresses, metrical positions, durations, phrasings, contours, and formal patterns of a poem to its musical realisation^1,35,36^. Compositional canons often entail tension being synchronised across the two domains (e.g., tense words realised on dissonant sonorities), though the full range of artistic expression emerges from exploring all possible combinations within style-specific constraints. Narrative structure in song lyrics unfolds through semantic and syntactic relations that evoke affective tension^37^. As in music, predictive expectations about the sequence of events described across sentences produce waves of suspense (tension). Narrative tension fluctuations are bound to predictive expectations about causative dimensions in the narrative plot – such as space, time, and characters^38^ – where the completion of causes and effects leads to tension release. Narrative divergences that impede causation completion and prolong tension produce measurable effects in brain activity^39,40^ and behaviour^41^.

While similar cognitive mechanisms have been proposed to underlie tension processing in both music and linguistic-narrative plots^42,43^, direct comparisons remain lacking. Our study takes an initial step, by considering perceived and rated tension in lyrics without imposing specific theoretical frameworks regarding local (semantic) or complete plotline (narrative) foundations. We namely explore how tension derived from paradigmatic features of music and language (tonal and semantic tension, henceforth M-tension and L-tension respectively) align to create affect during naturalistic listening of songs with lyrics. We aim to provide preliminary evidence bearing on the hypothesis that songs’ capacity to produce affect is – in a Gestalt sense – greater than the sum of their musical and linguistic parts, by considering how M&L building blocks converge as M- and L-tension. Specifically, we investigate whether affect formation in M&L, construed as a second-order property emerging from tension, demonstrates “superadditivity”, due likely to non-linear integration^44^. A simplifying proxy question is whether holistic (M&L) tension is *primarily* driven by the L- *or* the M-tension component. Our second hypothesis proposes that more musically sophisticated listeners would demonstrate enhanced ability to perceive tension *independently* for the two domains, as though uncoupled from one another.

In testing these hypotheses, we also conceptualised ratings in informational terms. Information is not monolithic: qualitatively different types exist, and many empirical settings allow disentangling redundant, unique, and synergistic informational components among source variables relative to a target variable^45^. Consider visual perception as a “target” example: each eye provides *unique* information about its peripheral field unavailable to the other eye. Overlap (redundancy) among them provides robustness. However, stereoscopic depth perception emerges only when both eyes process input together as synergistic information. In songs, redundant information likely emerges from alignment or congruency between musical events and their semantic realisation, constrained by genre-specific rules such as syllabic versus melismatic text-setting, or stress and accent alignment. This redundancy can enhance perceptual fluency and reinforce affective responses, while synergistic interactions between music and lyrics may contribute to emergent cross-domain aesthetic experiences exceeding the sum of their individual (single-domain) contributions. Finally, the construct of tension entails slow accumulation punctuated by moments of resolution and insight. Tension builds over extended timescales (tens of seconds to minutes), making experimental composed stimuli (typically under 10 seconds) too reductionist for our investigation. Within our chosen broad musical style (Western) and stimulus type (naturalistic songs with words), the requirement for wide ranges of elicited affective states in both M&L led us to select art songs from the Romanticism as an appropriate case study.

## Methods

### Participants

For the first two stimuli, thirty-four participants were recruited using the online platform *Prolific* (www.prolific.co). Being a native speaker of German (the language used in the stimuli) was the sole inclusion criterion, with no mention of musical education. After outlier removal (see section "Data preprocessing"), data from a total sample of *N*=28 participants remained available for analyses (average age 36.0 ± 14.3 years; 18 males). A second sample, of thirty-nine participants, was similarly recruited for the third, subsequently-added stimulus, of which *N*=35 remained after outlier removal (average age 37.3 ± 13.7 years; 24 males).

### Stimuli

Within the confines of the selected musical style, the choice of stimuli was based on inclusion/exclusion criteria (desirable/undesirable features), which we defined as follows:

- Tonal and textual content plausibly eliciting broad ranges of tension ratings in both dimensions;
- Monophonic or polyphonic texture, but avoiding strong melismas (large asynchronies between the voices’ syllabic content) to maximise comprehension;
- Relatively fast syllabic tempo, again to maximise comprehension and allow a good range of L-tension ratings;
- Duration maximum 3 min., to maintain listener attention under repeated listenings (experimental conditions);
- Avoid large variations in non-tonal dimensions, e.g. dynamics, metrical complexity;
- Avoid insofar as possible repetition of musical or textual content;
- Avoid text with excessive use of archaic, metaphoric, or religious language.

Some of the above criteria were also used in previous studies that, like us, focused on tonal as opposed to other types of musical tension (e.g.^46^). Our interest in music-structural (as opposed to extra-musical) contributors to tension entailed treating inevitably-remaining variation in non-tonal dimensions, such as rhythm and loudness, as nuisance variables. We used freely available recordings of the selected works: “Nachtlied” by Max Reger, “Letzte Bitte” by Hugo Wolf, and "O süßer Mai" by Johannes Brahms (see Supplementary Materials for scores).

### Task

The task was implemented using the *jsPsych* JavaScript framework^47^ and administered online using Pavlovia (www.pavlovia.org). Following a short practice trial to get participants accustomed to the online interface, we invited them to listen to the selected songs and rate perceived tension under three separate conditions, directing their attention to: tonal tension in the music alone (**M**), semantic/narrative tension in the lyrics alone (**L**), and holistic tension in the song as a whole (**M&L**). The order of songs, and of conditions within songs, was counterbalanced across participants. The exact wording of the instructions used in the task can be found in the Supplementary Materials.

**Fig. 1:**
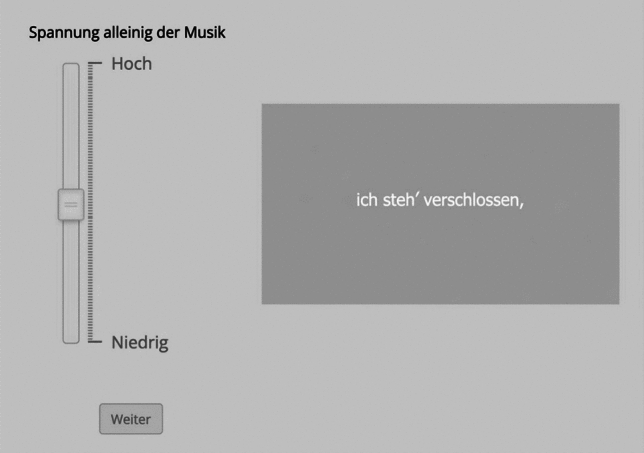
Online interface used in Pavlovia for collecting tension ratings, with a mouse-controlled slider on the left-hand side, going from low (“niedrig”) to high (“hoch”), and karaoke-style lyrics on the right-hand side (depicted is a line from "O süßer Mai" by Brahms). The instructions at the top solicited tension ratings as per the current condition (depicted: music-only condition, "Spannung alleinig der Musik").

A karaoke-style display was created for each stimulus, consisting of individual lines of text from the poem, largely following the original lineation (Fig. 1). Text between line breaks was held on the screen against a black background while the corresponding audio was heard. This was done to homogenise the portions of text under participants’ attention at any given time, notwithstanding any melismas or text asynchronies between voices. Participants were encouraged to move the on-screen slider continuously, using the mouse, to closely track their subjective tension rating. The slider sampled upon change rather than at a fixed sample rate, with data points – representing judgements standardised to a 0-100 scale – no less than 100 ms apart.

**Fig. 2:**
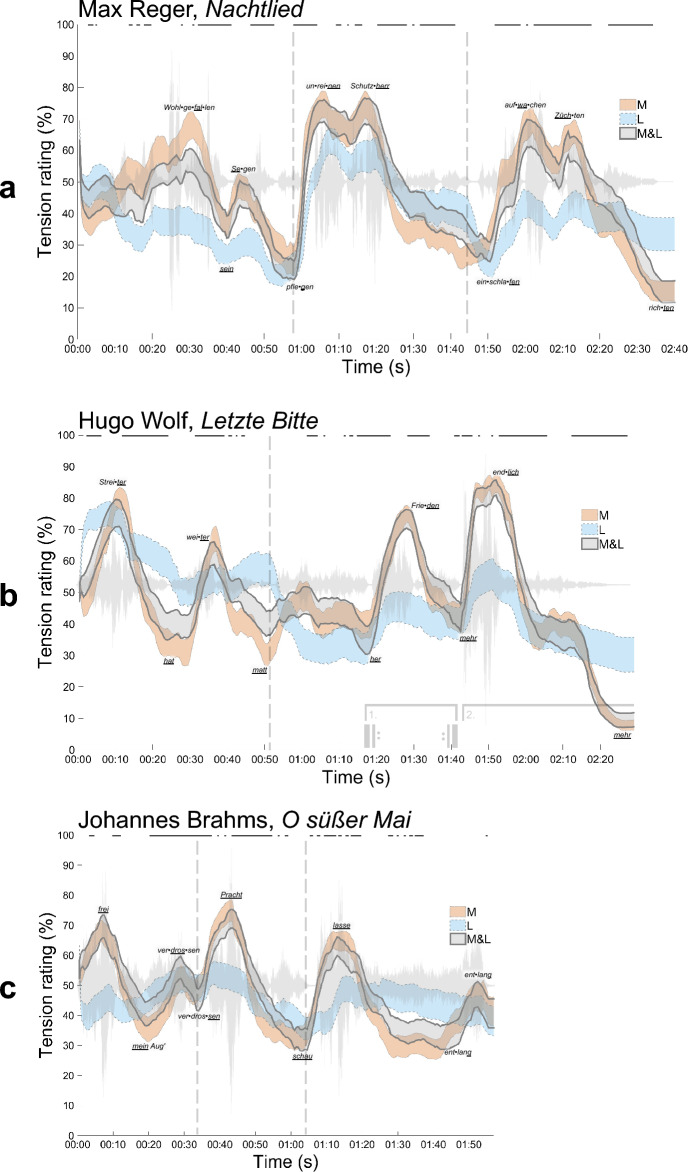
Mean tension ratings for the three stimuli used, under the three conditions (M: musical tension; L: semantic/narrative tension; M&L: holistic tension). Confidence bands denote ±1 SEMs (sample sizes: *N*=28 for Wolf and Reger, panels **a** and **b**; *N*=35 for Brahms, panel **c**). Plots do not change significantly if using medians ± SIQRs instead of means ± SEMs. Black horizontal lines at the top of each panel denote periods where mean M and L ratings simultaneously increase or decrease, i.e. when the difference signals are of the same sign. Vertical dotted lines approximately denote boundaries between stanzas. Words uttered close to prominent local minima/maxima of the M&L condition are annotated, with the specific syllable underlined (see Supplementary Materials for full lyrics). Repeat signs , indicate the second half of the second stanza in the Wolf piece (panel **b**). Each recording’s waveform, representing loudness/dynamics across time, is superimposed in light grey on the ratings. Units of time are equally scaled spatially across the three pieces, leading to different horizontal stretches due to different durations. For animated versions of these plots, please see videos in the repository at https://osf.io/uc5b3/.

Before rating, participants were administered an 18-items subset of the German-language version of the Goldsmiths Musical Sophistication Index questionnaire [GMSI; ^48^]. The items request agreement on a 1-7 scale with statements concerning the frequency and self-reported skill of one’s everyday musical activities. From this subset, individual scores were computed for GMSI’s *General Experience* factor (also referred to as *General Musical Sophistication*), with range 32-126 and data norm quartiles 67, 82, 92. The sample mean score was 63, median 64. A per-subject score was derived from questions Q38 and Q39 of the GMSI, which specifically probe liking of classical music. Separate analyses were carried out for top scorers on this metric (which we defined as scores 1-3 from the 1-7 GMSI scale). Figure S1 shows group-level average ratings as Fig. 2, but including this subsample only.

### Data preprocessing

Data was screened by plotting each subject’s rating data under all conditions. Based on this, "low effort participants" were defined by setting an arbitrary cut-off of 40s as a maximally-acceptable duration of an idling slider. On this basis, 6 listeners from the first sample (Wolf and Reger), and 5 from the second (Brahms), were excluded from the dataset as outliers. To enable data aggregation despite different rating paces and durations, following the methodology in Farbood’s study on musical tension^27^, as well as Schubert’s^49^ recommendations regarding continuous self-report measures, we resampled all subject judgements at 2 Hz (a data point every 500ms), using linear interpolation. A higher frequency was considered unnecessary given typical response times, and could lead to inflated number of observations.

Participants varied in terms of the slider range utilised during rating: some readily oscillated between the scale ends, while others hovered around restricted middle regions of the scale with minimal slider motion. We decided to treat this nuisance variation related to individual biases/differences of non-interest rather than actual differences in felt tension, normalising all curve samples to 0-100%, separately within each piece.

We used non-parametric statistics (Spearman rank correlation) to compare rating signals. Furthermore, since reporting correlation coefficients between raw time-series generally produces inflated results due to serial correlation^49^, we also considered in analyses the rating signals’ first derivative. Given the homogenous resampling, first derivatives amounted to a “difference” transformation amongst consecutive values in the time series. Further, we quantified directional congruency as the proportion of time where M and L ratings simultaneously increased or decreased, based on the signs of these difference signals, within 7s sliding windows with a tolerance of 1.5s in each half-window. These proportions amounted to 55%, 56%, and 47% of the total song time for the Reger, Wolf, and Brahms songs respectively.

All data preprocessing and analyses, including plot generation using custom scripts, were conducted in MATLAB (version R2023b; MathWorks, Natick, MA).

### Data analyses

#### Validation of L-tension ratings

We did a quantitative validation of L-tension ratings by considering maxima/minima words also in the L condition, where attention was specifically drawn to the lyrics. We did this by correlating, across words, the L-tension rating against the word’s score in relevant natural language processing and computational psycholinguistics databases. We namely used the *Berlin Affective Word List Reloaded* (BAWL–R), with 2,902 German words rated for valence, arousal, and imageability^50^; and the *Emotion Intensity Lexicon* [NRC-EIL;^51^], with 19,971 English words rated following Russell’s valence-arousal model^52^, manually identifying the closest English equivalent to each German maximum/minimum word (see data at https://osf.io/uc5b3). L-tension ratings positively correlated with the Arousal dimension of both databases (BAWL–R: *r*=.58, *p*<.05; NRC-EIL: *r*=.51, *p<*.05; see Figures S3 and S4).

#### Regression of holistic ratings on single-domain ratings

Ratings in the holistic (M&L) condition were submitted to multiple regression (*fitlm* function in MATLAB), with ratings from the M and L conditions as predictors of interest, including their interaction (M×L). To parcel out variance in the rating signals due to acoustic confounds, the loudness (amplitude envelope) of each song, extracted using v1.7.2 of the *MIR Toolbox* in MATLAB^53^, was added as a nuisance regressor (covariate). Given the high inter-subject agreements obtained previously for all conditions, the regression was computed on the averaged ratings, resampled so as to maintain equal numbers of data points.

Continuous rating signals naturally have high autocorrelation, whereas standard linear regression assumes independent errors. We therefore interpret the regression coefficients primarily as descriptive indicators of the relationships among the rating signals, and base our statistical inference on the surrogate-based null models used in the PID analysis (see below), which test whether the observed informational contributions exceed those expected by chance while preserving the temporal structure of the data. Accordingly, no correction for multiple comparisons was applied to the regression p-values, as these are not the basis for the paper’s inferential claims.

#### Information decomposition of holistic ratings

To operationalise the informational contributions of the single-domain rating signals to the holistic rating, we used Partial Information Decomposition (PID). PID is an information-theoretic framework that partitions the information that multiple source variables provide about a target variable into four components: unique information from each source, redundancy, and synergy^54^. We chose the MATLAB toolbox for model-free Partial Information Decomposition [mfPID; ^55^], an implementation that provides a model-free estimator suitable for continuous behavioural rating time series, and allows direct estimation of the four informational components.

For each song, average ratings were submitted to a procedure that estimates coefficients for all informational contributions. We used the difference-transformed signals (see “Data Preprocessing”) to maintain the dynamics of tension changes while partly mitigating the impact of these signals’ temporal autocorrelation. Ratings for the music-only (X_1_=**M**) and lyrics-only (X_2_=**L**) conditions were treated as source variables, and the averaged holistic rating (Y=**M&L**) as the target variable. The three time series were discretised into deciles using the mfPID_quantization function, then submitted to mfPID_2sources_discrete, which estimates the unique, redundant, and synergistic informational components of the decomposition. 

Statistical significance was assessed using 1,000 surrogate datasets obtained by circularly shifting the source time series relative to the target signal by a random interval between 10% and 90% of the total signal length. This desynchronisation disrupts temporal phase alignment among individual time series, while preserving their intrinsic autocorrelation (which is reduced but not eliminated by the difference transformation). For each informational component, the value observed in the original data was compared against the null distribution generated from the surrogates to obtain an empirical *p*-value^56^.

The MATLAB scripts used for the analysis are available in the OSF repository at https://osf.io/uc5b3.

#### Entropy

For each individual rating curve, we additionally computed the approximate entropy, a measure of the complexity of the time series, quantifying the degree of irregularity and unpredictability in its fluctuations by assessing how similar patterns of values remain across different time scales. We used the *approximateEntropy* function in v23.2 of MATLAB’s *Predictive Maintenance Toolbox*, which estimates the entropy of uniformly sampled time-domain signals by reconstructing the phase space. While commonly used to quantify predictability and regularity in biosignals, approximate entropy^57^ has also been shown to discriminate between clinical mood fluctuations based on self-reported ratings^58–60^. The resulting distributions were submitted to 1-way ANOVAs, separately for each piece, with condition (M, L, M&L) as a within-subjects factor.

### Consent and institutional approval

Participants were recruited online via *Prolific,* using native-language filters, and gave their informed consent before starting the experiment. The study was conducted according to the criteria of the Declaration of Helsinki (1964, amended in 2013), and its recruitment and data collection procedures were approved by the Ethical Committee of the University of Trento (Protocol nr. 2025-031ESA).

### Data availability

The repository at https://osf.io/uc5b3/ contains the raw data and the analysis scripts.

## Results

Figure 2 shows group-averaged ratings under each condition for the three songs. Words uttered around prominent maxima and minima of the M&L rating are highlighted in the plot. Visually, it can be observed that the M&L curves are generally more closely tracked by the M than by the L curves, a result we explored further with the analyses.

For each participant, an "M-L coupling" metric was computed to quantify how closely the rating curves under the M and L conditions respectively tracked each other, averaging across stimuli. This metric was operationalised as dynamic time warping (DTW) between the respective raw rating signals, with lower DTW values indicating more similarity. We then computed the Pearson correlation between this coupling metric and GMSI scores, across participants. This was non-significant (*p*=0.16, see Figure S2).

### Inter-subject agreement

For each song, rated under each condition, we assessed inter-subject agreement using ICCs (intra-class correlations). Given our data structure (multiple raters and measurements), the *ICC(2,k)* form was deemed suitable^61,62^, where higher values of *r* indicate greater inter-subject agreement or reliability in the ratings. This was computed using a custom MATLAB function (https://www.mathworks.com/matlabcentral/fileexchange/22099). Results are shown in Table 1.

**Table 1:** Intra-class correlations (ICCs). LB and UB represent the lower and upper bounds of confidence intervals for the *r* statistic of ICC(2,k), estimated for each song and each condition. The *F*-value, degrees of freedom and the corresponding *p*-value for the hypothesis test (*α*=0.05) are also reported.

**Song**	**Condition**	***r* for ICC(2,k)**	**LB**	**UB**	***F***	**df1**	**df2**	***p***
**1 (Reger)**	M	0.95	0.94	0.96	19.77	320	8640	<.001
	L	0.88	0.86	0.90	8.36	320	8640	<.001
	M & L	0.95	0.94	0.95	18.58	320	8640	<.001
**2 (Wolf)**	M	0.96	0.96	0.97	27.76	298	8046	<.001
	L	0.91	0.90	0.93	11.42	298	8046	<.001
	M & L	0.96	0.95	0.97	25.26	298	8046	<.001
**3 (Brahms)**	M	0.91	0.90	0.92	11.28	320	10880	<.001
	L	0.53	0.45	0.60	2.12	320	10880	<.001
	M & L	0.89	0.87	0.91	9.13	320	10880	<.001

There was *fair/good* inter-subject agreement in terms of the language rating, and *good/excellent* agreement in the music and the holistic ratings, following ICC benchmarks by Cicchetti & Sparrow^63^: <.5 *poor*; .5–.75: *fair*; .75 – .9: *good*; and >.9: *excellent* agreement. This justified henceforth using the average values in analyses.

### Regression of holistic ratings on single-domain ratings

As seen in Table 2, the M×L interaction term was only significant for the Reger piece, albeit with a small coefficient, suggesting little contribution to the holistic rating from the predictors’ interaction, beyond the single-domain contribution of each. This would suggest the holistic condition is merely a linear combination of the M and L conditions, with no evidence for a role for their interaction. Further, coefficients were significantly greater for M than for L in the Wolf (Wald test: *W*=13.96, *p*<.001) and Brahms pieces (*W*=2.08, *p*<.05); while the L coefficient was greatest in the Reger piece (pairwise Wald tests, all *p*s<.05). This is also suggested visually from the plots in Fig. 2.

**Table 2:** Multiple regression of holistic ratings on single-domain ratings, using difference scores. M and L represent the respective tension predictors, and M×L their interaction term. Separate results are shown for the "classically-inclined" subsample who expressed a preference for this musical style.

**Song / model**	**Term**	**All participants (*N*=28)**				**Classically-inclined subsample (*N*=7)**			
		Estimate	SE	*T*	*p*	Estimate	SE	*T*	*p*
**Reger**	(Intercept)	-0.06	0.04	-1.56	0.12	-0.06	0.07	-0.80	0.43
	M	0.49	0.03	16.38	<.001	0.43	0.04	11.70	<.001
	L	0.51	0.05	9.51	<.001	0.23	0.06	3.50	<.001
	M × L	0.05	0.01	3.55	<.001	0.03	0.03	1.10	0.27
**Wolf**	(Intercept)	-0.03	0.05	-0.75	0.46	-0.04	0.09	-0.40	0.69
	M	0.55	0.03	19.87	<.001	0.39	0.04	8.74	<.001
	L	0.36	0.06	5.68	<.001	0.17	0.08	2.17	0.03
	M × L	0.02	0.02	0.98	0.33	-0.03	0.02	-1.35	0.18
**Brahms**	(Intercept)	-0.03	0.03	-0.77	0.44	-0.03	0.08	-0.38	0.71
	M	0.63	0.03	18.25	<.001	0.28	0.07	4.23	<.001
	L	-0.02	0.07	-0.36	0.72	-0.28	0.10	-2.91	<.001
	M × L	0.04	0.04	1.01	0.32	-0.17	0.07	-2.54	0.01

Finally, when considering only the small "classically-inclined" subsample, the interaction term was significant only for the Brahms piece with a larger coefficient, suggesting in this case the holistic rating is a less straightforward, possibly synergistic, combination of the single-domain ratings. As overall the regression results did not speak clearly to our main hypothesis, we explored the data further with an information-theoretic analysis, described next.

### Information decomposition of holistic ratings

Under the circular-shift null distribution, the PID analysis returned significant coefficients for all informational components in the Reger piece only, and for the M-unique component in the Wolf piece (see Table 3). The interpretation of these components in relation to the musical and textual features of each piece is taken up in the Discussion and in the Supplementary Materials. An earlier pointwise shuffle procedure, which does not preserve signal autocorrelation, yielded broader significance across pieces but is not reported, as the circular-shift procedure is adopted as the primary analysis throughout.

**Table 3:** Partial Information Decomposition (PID) results, with the M (music) and L (language) ratings as sources (U1 and U2 respectively) and the M&L rating as target. We report the coefficients for the unique (U), redundant (R), and synergistic (S) information contributions. PID components whose values exceeded the surrogate-data threshold (α = .05, corrected for multiple comparisons) are in bold letters.

	**U**_**1**_** (Unique|Music)**	**U**_**2**_** (Unique|Lyrics)**	**R (Redundant)**	**S (Synergistic)**
**Reger**	**0.034** **(*p*<.001)**	**0.003** **(*p*=.031)**	**0.042** **(*p*<.001)**	**0.006** **(*p*=.002)**
**Wolf**	**0.012** **(*p*=.024)**	0.000 (*p*=1.000)	0.000 (*p*=1.000)	0.000 (*p*=.995)
**Brahms**	0.000 (*p*=1.000)	0.000 (*p*=1.000)	0.000 (*p*=1.000)	0.000 (*p*=1.000)

The greater unique coefficients for M than for L in the Reger piece suggests that, as also seen in the regression, taken in isolation the music alone explains a higher percentage of the variance in the holistic condition (which it indeed closely tracks) than the lyrics alone do. The significance of the L-unique component may, on the other hand, reflect the high variability seen in that piece’s L-tension (see Discussion).

Although the interaction terms in the regression analysis were small or non-significant, we consider the PID results more reliable because PID explicitly distinguishes between redundant, unique, and synergistic contributions of predictor variables to the holistic tension ratings, rather than assuming a strictly additive or linear relationship. Unlike standard regression, which tests for linear interactions and may overlook non-linear dependencies^64,65^, PID can capture higher-order interactions that arise from the combined effect of music and lyrics beyond their individual contributions^45,54^. This enables PID to take into account the differing roles that the single-domain L rating had in each of the pieces in a more nuanced and theoretically justified way.

### Entropy

**Fig. 3:**
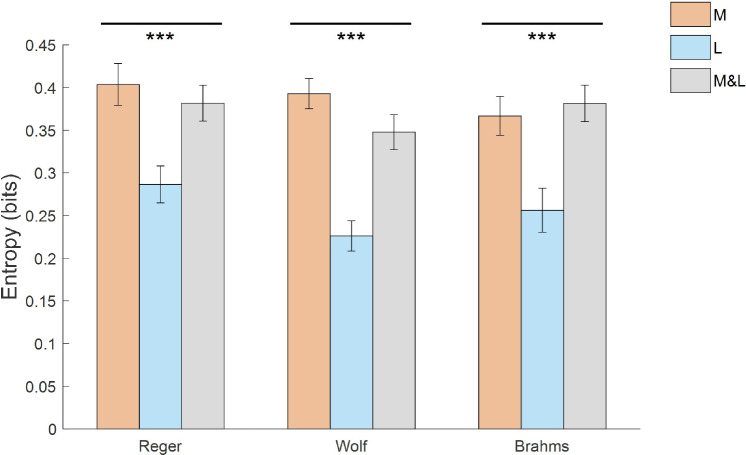
Distribution of entropy values corresponding to individual rating curves. The main effect of condition is significant for all pieces. Error bars show ±1 SEMs.

The main effect of condition (M, L, M&L) was significant for all pieces (all *p*s<.001), as seen in Fig. 3. Follow-up Bonferroni pairwise comparisons revealed significant differences between L and the remaining two conditions (all *p*s<.05). These results suggest tension dynamics are more predictable when only lyrics are considered.

## Discussion

In this study, we explored how different dimensions of tension interact during real-time naturalistic listening of choral music. We tested the hypothesis that the integration of music and lyrics is synergistic, utilizing a Partial Information Decomposition (PID) framework to analyse the relationship between domain-specific ratings – tonal tension in music and semantic tension in lyrics – and holistic ratings collected under holistic listening instructions. Our results revealed a significant synergistic component for the Reger piece, lending tentative support to our hypothesis and suggesting that the tight coupling of musical and linguistic features can drive superadditive affective experiences. Beyond the specific idiom of late-Romantic word-painting, these findings suggest that the experience of tension serves as a primary ‘currency’ for cross-domain integration. This information-theoretic framework may provide a scalable approach for exploring how musical and semantic features interact to shape the emotional trajectory of vocal music. While our current findings are genre-and even stimulus-specific, they may provide a proof of concept for the existence of synergistic integration in music more broadly, and perhaps even across diverse information streams (e.g., music and movement in dance, or music and moving images in cinematography).

### Holistic ratings more closely tracked by M than by L ratings

Consistent with the descriptive regression results, holistic ratings were more closely aligned with M- than with L-tension ratings, suggesting that moment-to-moment affective experiences in song are primarily driven by the musical dimension. Evident in the greater regression coefficients for the M than the L term, this finding supports an intuition that, at least for the selected musical pieces and style, listeners do not consciously isolate lyrics but instead experience an overall affect largely dictated by the musical dimension. However, this may vary with musical style and individual factors, including cultural background (see next section).Contrary to our expectations, more musical listeners did not show stronger independent appraisal of M- and L-tension, as our M-L coupling metric was not predicted by GMSI scores.

This finding aligns with differences in the temporal granularity at which events are attended to in music versus in language. Instrumental music and speech differ in production rates^66^ and modulation spectra^67^. However, the case of sung music with lyrics has important particularities. Indeed, many paradigmatic differences between M&L (“design features”,^68^) entail differences between spectrotemporal features of their vocalised forms. For instance, critical events occur faster in regular speech than in song^69^. However, sung words often unfold at an even slower rate than the corresponding (note-level) musical events, particularly in our chosen style where, despite our stimulus selection seeking to avoid melismas, sung phonemes are typically elongated across musical measures. In our data, this difference in temporal granularity was reflected in lower approximate entropy in the L condition, where only lyrics were considered, presumably leading to more predictable tension dynamics. The slow pace of the lyrics may have hindered the emergence of discernible narrative arcs (e.g.^70^) in those tension curves. Approximate entropy has previously been applied to self-reported data in contexts different from ours, e.g. as a marker of mood changes in psychiatric populations^59,60,71^. Nonetheless, we regard this finding as consistent with our main result expressed in terms of information decomposition of the rating signals.

Inter-subject agreement was higher for music and holistic ratings than for lyrics ratings across all three pieces, with the Brahms L-tension ratings showing borderline-low agreement (ICC = 0.53). This pattern supports the notion that tonal rather than semantic elements predominantly shape group-level appraisal of tension, probably through reduced ambiguity – and suggests that the role of lyrics in driving tension may be especially variable across listeners in pieces where the lyrical pacing is slow and the narrative arc less salient. Our validation of L-tension ratings against affective word databases bolsters our confidence in the robustness of this dimension of the rating data and hence in the conclusions concerning it – with a few caveats. Since lyrics are processed in context, broader analytical windows (e.g., phrase-level instead of isolated words) might have yielded even stronger correlations. For example, in the Reger piece, it was likely “unreinen Geister” that was responded to, whereas our analysis based on local minima and maxima necessarily had to work with word ratings for either “unreinen” or “Geister”, each with rather different database scores, potentially introducing noise. Moment-by-moment rating instructions may have further constrained more natural engagement with lyrics, that is, within longer windows of temporal integration, limiting their perceived influence on tension (see related note under Limitations).

### Cross-individual and cross-cultural variation in the perception of song narrative

Shared musical experiences foster shared perceptual representations and possibly even shared internal representations^72^. When imagining stories while listening to instrumental music, listeners consistently associate certain event types with certain musical features, e.g. an attack scene upon a sudden increase in dynamics^73^. This suggests a continuum of specificity in music-evoked associations, from purely musical features, e.g. chromaticism or diatonicism, to purely extra-musical references, as found e.g. in popular culture^74^.

Music’s expressive effects^75^, particularly tension-relaxation dynamics^76^, can evoke imagined narratives even in the absence of lyrics. McAuley et al.^77^ demonstrated that US and Chinese listeners reliably matched wordless musical excerpts to culture-specific stories, indicating that music without lyrics is not an abstract, asemantic medium, but instead one that engenders concrete narrative perceptions, shared by co-enculturated listeners. The high inter-subject consistency in our M-tension ratings in our study does not rule out a similar quasi-semantic mechanism, where our German-speaking participants may have implicitly engaged with a latent narrative even when instructed to ignore lyrics. Our songs’ lyrics, less suggestive of an explicit narrative, might paradoxically have left more room for individual interpretive variability than had words been absent, leading to the reduced L-tension rating consistency (relative to M-tension).

### Correspondences between ratings and song structural features

L-tension ratings tracked M- (and therefore holistic) ones most closely in the Reger piece, as confirmed by pairwise comparisons among regression coefficients. It might be that the affective load of lyrics in the Reger piece was more synchronously-distributed with the tonal tension, leading to a more balanced weighting of M and L as drivers of holistic rating. Additionally, directional congruency between M- and L-tension was more common in the Reger and Wolf pieces than in the Brahms piece, reflecting varying “phase differences” between the realisation of musical and textual events in the text-setting of each piece. The Brahms piece, with slower lyrical pacing, exhibited lower inter-subject agreement in L-tension ratings, possibly indicating a qualitatively distinct relationship between music and text.

A cross-correlation analysis of the difference-transformed M and L signals further characterised the temporal relationship between the two rating curves. Across all three pieces, peak cross-correlations occurred within ±500ms of zero lag — the resolution limit of the 2Hz sampling rate — suggesting that M- and L-tension dynamics are broadly temporally aligned at the timescale captured by our paradigm, consistent with the established literature on response lags of 1–3 seconds in continuous tension rating tasks^28,78^. Nonetheless, the directional tendencies differed across pieces in ways that cohere with their musicological profiles. Namely, for the Reger, the two signals were maximally aligned at zero lag, consistent with the tight M-L coupling evidenced by its PID results (see Results) and the frequent coincidence of tonal and semantic emphases in that piece’s text-setting; for the Wolf, M marginally preceded L, consistent with the primarily tonal-dynamic drivers of tension in that piece; and for the Brahms, the near-zero lag was directionally ambiguous and is therefore not interpreted further.

The convergence of significant redundant and synergistic PID components specifically in the Reger piece is consistent with features of that work identified in our musicological analysis (Supplementary Materials). The redundant component suggests that M- and L-tension share predictive information about holistic tension – plausibly reflecting the tight coupling between musical and textual affect in a piece characterised by dense chromaticism and homophonic texture, where tonal and semantic emphases frequently coincide. The synergistic component further indicates that the holistic tension ratings contain information about the combined music-lyrics signal not present in either source alone – that is, music and lyrics interact rather than merely co-occur in shaping the listener’s response. If our interpretation is correct, then lyrics, while not primary drivers, contribute at least as congruency cues (redundant information) alongside tonal elements, and their combination with music generates emergent affective information under holistic listening conditions.

We sought to link musical and textual features to rating curve maxima and minima, acknowledging inherent response lags between the hearing of a musical event and the listener adjusting the slider in response. Previous work that modelled individual chords equally-spaced throughout a piece, could achieve this by segmenting rating time-series into delay and stable regions to account for motor preparation and execution at chord-level^79^. However, such models assume that tension remains unchanged during sustained chords, which may not hold in naturalistic listening. Since we did not model individual events, for the score-based interpretation we resorted to a subjective, case-by-case account of the delay between points on the rating curve and their associated score events, detailed in the Supplementary Materials.

While our primary interpretation focused on the informational relationships among tonal and semantic features, a complementary perspective concerns the role of attentional mechanisms in shaping listeners’ judgments. Our experimental manipulation consisted essentially of instructing listeners to shift their focus of attention toward musical features, semantic content, or the song as a whole. In this view, attentional allocation does not replace the feature-based interpretation, but provides a mechanism by which listeners may differentially weight information from the musical and textual domains. Previous work suggests that directing attention to different musical aspects can modulate perceived tension in systematic ways (e.g.^80^). Selective attention to a single domain (music or lyrics) may thus enhance the *unique* informational contribution of that source to the listener’s judgment, whereas attending to the song holistically may promote integrative processing of cues from both domains. In the PID literature, such integrative contributions are sometimes illustrated using basic logical analogies, such as the XOR operator, whose outcome necessarily depends on the joint state of multiple inputs rather than on either input alone; and is thereby naturally associated with the *synergistic* component^81^. In the present context, this analogy maps not onto the task structure itself but to the estimated *synergistic* PID component, which reflects information emerging from the combined influence of musical and semantic cues under holistic listening conditions. Although the present study does not directly measure the neural mechanisms underlying such attentional shifts, framing the results in terms of attentional selection may help connect naturalistic behavioural paradigms such as the one we used to mechanistic accounts of attention from cognitive neuroscience^82^. Future work combining behavioural and neurophysiological approaches may help clarify how attentional mechanisms contribute to the integration of music and language during song listening.

Taken together, these findings suggest that the integration of musical and linguistic tension during song listening depends not only on the individual contributions of each domain but also on how their informational structures align within specific musical contexts.

### Limitations and future directions

Ours was a phenomenal approach to tension as a high-level phenomenon, summarising rather than mechanistically explaining it^83^. This necessarily entailed both obscuring fine-grained interactions and remaining agnostic to song-specific “tactics” of tension manipulation that would ideally be invoked to explain the data. For instance, we sought to minimise textual repetition in stimulus selection, as our paradigm was not suited for analysing its effects^84,85^. Nonetheless, some repetition occurred in the Wolf piece, though partially mitigated by the karaoke format showing one row at a time. However, a thorough treatment of the topic of tension build-up in complex vocal music will need to theoretically and empirically account for multi-level repetition, arguably as crucial an aspect for song lyrics as duality of patterning is for language in general^86^. This focus on high-level phenomena acknowledges a broader trade-off, as we explain below.

Our analyses remain necessarily distanced from the more granular, song-specific complexities – such as intricate harmonic and rhythmic dependencies, or sophisticated text-setting devices – that define the unique expressive architecture of these compositions. The present framework also does not formally model the latent cognitive transformations through which these compositional nuances are integrated by the listener before manifesting as the high-level tension ratings analysed here. Even within our relatively restricted stylistic sample, the three pieces differ markedly in how they distribute and coordinate affective content across music and lyrics — in terms of lyrical pacing, M-L temporal alignment, and the balance of tonal versus text-driven tension. This within-sample heterogeneity itself cautions against treating our findings as representative even of late-Romantic choral song as a genre, let alone beyond it; and underscores the need to interpret the piece-specific results with appropriate caution.

We took choral music of the late Romanticism as a fertile “model organism” to isolate the interaction between perceived tension in the domains of M&L. While our stimuli were drawn from this specific tradition, they represent only a subset of the vast idiomatic strategies found across the vocal repertoire. Clearly, with the present data we cannot claim external validity beyond this style. The finer-grained mechanisms of M&L integration, though possibly sharing some neural code^14,87,88^ (but see also^89^), undoubtedly find different implementations, reflecting diverse compositional strategies employed across different styles of vocal music. In particular, the role of lyrics in determining affect in songs likely entails a non-linear relation to style-specific characteristics. For example, slow syllabic rates in Russian sacred music may still strongly influence tension appraisal, probably by lengthening the window of temporal integration of M&L features, although this influence is likely challenging to assess in live-rating tasks. Nonetheless, some mechanisms we identified may plausibly generalise across cultures: first, tension-relaxation patterns are a device common to many non-Western musical traditions^1,90^. Second, developmental and cross-cultural work shows that infants and unenculturated adults arrive at convergent internal representations of musical meter^91^ and musical phrasing^92^, and inferences about song function^93^. Together, these convergences imply at least some biologically-based universal underpinnings in humans’ cognition of music^94^.

In sum, future research should combine cognitive neuroscience insights on M&L feature integration with music-analytical insights into their aesthetic functions. Specifically, neurophysiological measures can track real-time integration processes, to more closely specify the role of temporal granularity in M- and L-tension appraisal. Such studies could bridge top-down modelling approaches from music theory, such as those by Farbood^27,79^ and Lerdahl et al.^26^, with bottom-up models based on automatically extracted musical features such as Barchet et al’s^95^ predictive model calibrated for Western classical music. Expanding stimulus selection to diverse musical styles, and combining the ecological validity of naturalistic listening with independent manipulations of M- and L-tension in bespoke recorded stimuli, would further advance this research. For instance, future work could complement this naturalistic paradigm by employing experimentally manipulated stimuli that selectively isolate musical and linguistic components, allowing a more reductionist investigation of how these cues contribute to perceived tension.

### Conclusions

We aimed to quantitatively explore the interplay between musical and linguistic tension in shaping affective experiences in song listening. The tonal dimension emerged as the dominant driver of holistic tension appraisal, though lyrics also played a significant, albeit variable, role across pieces. For the Reger piece, the significant redundant and synergistic informational components suggested that musical and semantic tension are not merely additive – their combination carries information about holistic tension beyond what either domain contributes alone. Albeit based on a single piece from our stimulus set, and thereby subject to replication and extension by future research, this finding lends empirical support to a long-held compositional intuition. More broadly, our results contribute to the understanding of cross-domain integration in human cognition and aesthetic experience, suggesting that predictive mechanisms operating across music and language jointly shape emotional responses to song. They also provide a foundation for further enquiry into cultural and individual differences in tension perception, and into how tonal and poetic structure coalesce to produce immersive affective narratives. Ultimately, such a line of work offers a promising path toward a unified understanding of how the human mind synthesises complex, concurrent information streams into a singular, cohesive experience.

## Supplementary Information

Supplementary Information 1.

Supplementary Video 1.

Supplementary Video 2.

Supplementary Video 3.

## Data Availability

The repository at https://osf.io/uc5b3/ contains the raw data and the analysis scripts.
