# Supplementary material for: Synergistic tension among the music and lyrics of late-Romantic choral songs
Source: Sci Rep. 2026 May 29;16:24576. doi: 10.1038/s41598-026-50356-x (PMC13454584; doi:10.1038/s41598-026-50356-x)
Supplement: Supplementary file 1 [file 41598_2026_50356_MOESM1_ESM.docx]

**Synergistic tension among the music and lyrics of
late-Romantic choral songs**

– Supplementary Materials –

Tudor Popescu^[1]^*, Adrian Kempf^[2]^, Patrick Boenke^[3]^, Marco Tettamanti^[4],^^[5]^

# 1. Supplementary results

Figure S1: Average tension ratings for the three stimuli used, under the three conditions (M: tonal tension; L: narrative tension in language; M&L: holistic tension), including only the "classicaly-inclined" subsample of participants who reported to enjoy classical music (*N*=7 for Wolf and Reger, *N*=8 for Brahms; see main manuscript; also see Figure 2 in the main manuscript, which includes the entire sample).
Confidence bands denote ±1 standard errors around the mean for each data point (*N*=28 participants for Wolf and Reger, panels a and b; *N*=35 for Brahms, panel c). Black horizontal lines at the top of each panel denote periods where M and L simultaneously increase or decrease, i.e. when the difference signals are of the same sign for these conditions (see text for details). Vertical dotted lines show approximate timing of the boundaries between stanzas. For ease of following, words uttered close to prominent local minima/maxima of the M&L condition are annotated, with the specific uttered syllable underlined (see below for full lyrics). Repeat signs are shown in panel b, corresponding to the first and second repetitions of the second half of the second stanza. The recording waveform, illustrating loudness, is shown above in each case. Units of time are equally stretched (occupy an equal amount of horizontal pixels) for all three pieces

Figure S2: Non-significant correlation between GMSI scores and a metric of M-L coupling (dynamic time warping). The coloured band shows 95% confidence intervals around the regression line.

Figure S3: Correlations between L-tension ratings at peak/trough words and those words' scores as coded in the BAWL–R, pooled across all three pieces. The critical correlation is the one between our tension ratings and the Arousal database scores (2^nd^ row, 4^th^ column).

Figure S4: As above but with word scores as coded in the NRC-EIL database.

# 2. Further links between ratings and structural (musical and textual) features of stimuli

The timepoints (mm:ss) refer to the recordings used for each of the selected stimuli.

## 2.1. Reger: "Nachtlied"

At the beginning of the song, M-tension shows an increase in stages, that culminates at ~00:33 (mm. 1 to 9 of the score). From a harmonic perspective, the song begins with some uncertainty, wavering between B minor (see the first chord) and D major at the end of the first phrase in m. 2. The second phrase (mm. 3–4) ends for the first time with a full formal cadence that gives greater prominence to F-sharp minor in m. 4. The third phrase, by contrast, is more expansive (mm. 5–9), contains more chromatically altered notes, and ends with an elaborate formal cadence in A major (m. 9), the dominant of the song's main key, D major.

It seems plausible that the overarching harmonic step progression I-III-V, directed toward the dominant A major, along with a process of increasing chromaticism, are (at least) partly responsible for the perceived tension process. In any case, the climax at ~00:33 coincides with an expressively embellished diminished seventh chord (m. 8, third beat) that initiates the following cadence as a predominant.

The section from mm. 10–14 (00:38–01:00) leads back to the tonic D major (with a previous short stop on B major in m. 12) and affirms the tonic for the first time with a formal cadence. It seems obvious that this process of "coming home", supported by the melodic movement to a lower register (especially in the soprano) and the retardation in tempo (m. 13), is associated with a decrease in tension, finally leading to a valley at the end of the cadence in m. 14 (00:58). The brief increase around 00:45, however, may be related to a gestural moment, i.e. to the sudden upswing of the soprano and alto voices in m. 11, expressing a certain emphasis.

The sudden increase in tension in the third beat of m. 14 (01:02) may be due to a moment of surprise: all voices begin simultaneously in the higher register and in forte. The now strictly homophonic structure, peaking in surprise and tension, places particular emphasis on the key sentence “Treib, Herr, von uns fern die unreinen Geister.” It is remarkable that the high level of (both M- and L-)tension is maintained until m. 20 (01:23). This may be due to the dwelling in the high register, the increasing chromatic complication of the chords (especially in m. 19), but also the increased dynamic level. The brief decrease in tension at 01:14 is likely related to a lighter cadence to F-sharp major (m. 18, third beat) and the stop on a longer note value (half note).

The passage from m. 21 to 26 (01:23 to 01:44) shows a continuous decrease in tension. This could be due to the reduction of dynamics, melodic downward motion (see again the soprano), less complicated harmony, and slowing of the tempo (m. 25).

In the last third of the song, a further increase in tension in a relatively short time is noticeable. The first impulse is most likely related to the melodic upswing in the soprano voice (mm. 28–29, 01:53–01:58). The second impulse around at 02:01 could be related to another moment of surprise. After the preceding stop on B major (m. 29), the entry on C major (m. 30) seems very surprising and is reminiscent of the chord progression of a so-called deceptive cadence.

Consistent with this tight integration of musical and textual events, a cross-correlation of the M and L difference signals for this piece yielded a peak at zero lag, suggesting that tonal and semantic tension dynamics are maximally temporally aligned — in keeping with the homophonic texture and synchronous text-setting that characterise this work.

## 2.2. Wolf: "Letzte Bitte"

*NB: The version shown in the scores below has been transposed to B minor (two sharps as accidentals in the key signature) for ease of reading. The text below refers to chords relative to the original key of the piece, B flat minor (five flats in the key signature).*

Four peaks are observed, each of which is reached after a shorter period of increasing tension: 0:11, 0:37, 1:29 and 1:46. These maxima are probably not exclusively, but very much related to the dynamic development, i.e., changes in loudness (piano to forte). The first three of those are namely at the end of crescendo movements, which are also very strongly worked out by the choir in the selected recording.

In 0:11 and 0:37, the harmony likely plays a role in increasing tension. Both peaks come at the end of two-measure phrases (in the score, mm. 1-2 and 5-6) whose harmonics are developed over a chromatically falling bass line. Such forms of chromaticism have been considered "pain-affecting" for centuries and are consequently associated with tension. Furthermore, the chosen sounds over the chromatic bass line are anything but conventional, and therefore not "predictable" for the listener which could also support the impression of tension increase. That the peak in 0:37 is lower than in 0:11 could be related to the fact that mm. 5-6 are a varied repetition of mm. 1-2, and thus bring to the ear a harmonic development that was similarly performed shortly before. It is also in a lower register, suggestive of lower tension.

In 1:29 and 1:46, the culmination has mostly to do with volume changes, rather than harmonic features. Immediately before 1:29, the harmony is rather static and conventional: a G-flat major chord (m. 13) is "composed out", i.e. unfolded in time. There is only one chromatic passing note (D natural in the soprano and alto line in m. 14) which could induce tension in a harmonic way. But this can likely not explain the very strong increase before 1:29, a development more probably related to the very strong dynamic increase.

In 1:46 (m. 17) there is no harmonic development targeting this point. It is the beginning of a new phrase, but it begins unpredictably in a sudden forte and with a very wide-spread chord (the soprano has its highest note so far at this point). Very likely, the tension felt here mainly has to do with these two "surprises".

Consistent with the primarily tonal-dynamic character of the tension peaks described above, a cross-correlation of the M and L difference signals yielded a peak at +500ms (M leading L), suggesting that musical events marginally precede the corresponding semantic tension response in this piece.

## 2.3. Brahms: "O süßer Mai"

The Brahms song is divided into three stanzas, the second and third of which are musical variants of the first.

The first stanza (mm. 1–11) is marked by colourful harmonic chord progressions, of which the entry of F minor (m. 7) after a preceding phrase ending in D minor (m. 6) is probably the most striking. With regard to D minor as a local centre the F minor on which "mein Aug'" is realised is suggestive of an inward gaze into the lyrical self (Kaiser, 2018). The lead of the first phrase (mm. 1–2) to the dominant builds harmonic tension, which intensifies till the beginning of m. 3 (00:08). With the soprano entry on the note e2 (m. 3), the highest note so far is reached, which may explain the M-tension peak at this position. This begins the resolution, and tension decreases till the valley at m. 6 (00:19). Measures 5–6 take over the motif of mm. 3–4 (see soprano). The transposition of the motif by a whole tone downward, the dynamic reduction and the darkening of the minor after the bright major evidently lead to a decrease in M-tension. Consequently, the valley coincides with the end of the phrase in m. 6 (third beat). The following rise in tension may be due to a rhythmic feature. In mm. 7–9, the motif from mm. 3–4 and 5–6 is taken up again, but now in augmented form, i.e. with enlarged note values. The resulting stagnation of movement is likely to be perceived as increasing tension, which we indeed see in the M-tension curve. The peak at 00:28 coincides with the minor subdominant in m. 10, which prepares a formal half cadence.

Due to the expectations set up in the first stanza, the setting of the 2nd stanza (in the original key of C major) appears bright and non-threatening, and functions as a contrasting B section (Kaiser, 2018). This stanza (mm. 12–21) is characterised by contrast, not harmonically but in terms of the rhythmic movement, onomatopoeically suggestive of a gentle breeze through the green blossoms of May. In the first half of the second stanza the tension curve is similar to the first, but with greater amplitude in both the opening increase and the following decrease. In the increase phase (beginning of the stanza), this could be related to the greater activity of the voices due to the use of faster (more granular) rhythmic values. Against this background, the rhythmic calming from m. 17 onward (00:42, after "Pracht") is likewise more pronounced. As in m.3 of the first stanza, the peak coincides with the soprano entry on note e2 in m. 14 respectively 15 (00:43). In difference to the first stanza, the motif sequences in mm. 17–18 and 19–21 now both appear in slightly stretched form, and thereby perceived as stages in a continuous resolution of tension, which reaches a valley point at the half cadence in m. 21 (01:04) without any further increase in tension as seen at the end of the first stanza.

The third stanza has the effect of a reprise or shortened recapitulation followed by a coda, thereby establishing the three-part form of the song, namely A-B-A with a coda (Kaiser, 2018). This stanza likewise shows a tension profile comparable to the first two, with a peak again on the soprano entry on the note e2 (m. 25, 01:13). The rise in tension at the end of the song is probably related to the ascending movement of the voices, which in the soprano reaches back to e2 (m. 37, corresponding to the peak seen in the data at 01:51).

A cross-correlation of the M and L difference signals yielded a peak within the resolution limit of the sampling rate (±500ms) and is not interpreted further given the ambiguity of the directional tendency relative to the other evidence for this piece.

# 3. Wording of task instructions (translated to English)

Hi, thanks for taking part in our musical tension study. Please make sure you are undisturbed for the next 10 minutes that we estimate this study will take.

Please wear headphones or earphones so as to hear the music in good conditions.

Your task is to rate tension in real-time, as you listen to a song we've selected, namely <song name here>. By "tension" we mean the degree to which the music, at a set point in time, gives you a feeling of tensing up (increased anticipation and perhaps anxiety about what comes next), versus relaxing (decreased anticipation/anxiety).

Note that this is distinct from your enjoyment of the music, as peaks in tension may or may not be enjoyable.

Please continuously move the on-screen slider with your mouse as the music plays, to indicate your tension going up/down.

The song you will hear has words. We will ask that, across three separate listenings (which together make up this study) you rate the tension you perceive in the *music* alone, the tension in the *lyrics (words)* alone, and finally the *overall* feeling of tension that you get as a whole when listening to the song as an entity. That means paying increased attention to the melody and to the words, and then to both equally.

You will get the 3 listening conditions in random order, over the next screens.

You are finished with the nth listening. You can take a few moments' break, then press Next to condition to the n+1th listening.

That was the study – thanks again for taking part. Press Next to exit.

# 4. Video legends (OSF repository; animated versions of Fig. 2)

The OSF repository (https://osf.io/uc5b3/) contains three videos showing animated versions of Fig. 2 (mean tension ratings), one for each stimulus. Their corresponding captions are provided below.

Video S1. **Continuous tension ratings for the Reger stimulus.** Animated trajectories of mean tension ratings under the three listening conditions (music-only, M; lyrics-only, L; and holistic, M&L) are shown over time, aligned with the unfolding audio stimulus.

Video S2. **Continuous tension ratings for the Wolf stimulus.** Animated trajectories of mean tension ratings under the three listening conditions (music-only, M; lyrics-only, L; and holistic, M&L) are shown over time, aligned with the unfolding audio stimulus.

Video S3. **Continuous tension ratings for the Brahms stimulus.** Animated trajectories of mean tension ratings under the three listening conditions (music-only, M; lyrics-only, L; and holistic, M&L) are shown over time, aligned with the unfolding audio stimulus.

# 5. Song texts and scores

NB: in the original-language version, syllables printed in red correspond to prominent peaks (maxima) in the average rating curve for the M&L condition, given the recording used to elicit ratings. Similarly, blue is used for valleys (minima) in that curve. See also the analyses done on the L-tension ratings.

Recordings available on YouTube were used, with no intention of copyright infringement.

## 5.1. Reger: "Nachtlied"

From ''Acht geistliche Gesänge'' op. 138

Composed: 1914

Voicing: SATBB

Text: Petrus Herbert

Composer: Max Reger

Recording / performer: Singer Pur

Recording duration: 2:40

| Die Nacht ist kommen,  Drin wir ruhen sollen;  Gott wal'ts, zum Frommen  Nach sein'm Wohlgefallen,  Daß wir uns legen  In sein'm G'leit und Segen,  Der Ruh' zu pflegen.  Treib, Herr, von uns fern  Die unreinen Geister,  Halt die Nachtwach' gern,  Sei selbst unser Schutzherr,  Beschirm Leib und Seel'  Unter deinen Flügeln,  Send' uns dein' Engel!  Laß uns einschlafen  Mit guten Gedanken,  Fröhlich aufwachen  Und von dir nicht wanken;  Laß uns mit Züchten  Unser Tun und Dichten  Zu dein'm Preis richten! | The night has fallen,  And we should rest;  God is there, to care for us  By his good will,  So that we settle  In his company and blessing,  To maintain the peace.  Father, drive the evil spirits  Far away from us;  Keep the night watch;  Be our protector;  Shield both body and soul  Under your wings;  Send us your angels!  Let us go to sleep  With good thoughts,  Happily awaken  And never waver from you;  Let us, with rearing,  Focus our deeds and words  On your glory! |
| --- | --- |

## 5.2. Wolf: "Letzte Bitte"

From ''Sechs geistliche Gesänge''

Composed: 1881

Voicing: SATB

Text: Joseph v. Eichendorff

Composer: Hugo Wolf

Recording / performer: Netherlands Chamber Choir, conducted by Uwe Gronostay

Recording duration: 2:29

| Wie ein todeswunder Streiter,  der den Weg verloren hat,  schwank' ich nun und kann nicht weiter,  von dem Leben sterbensmatt.  Nacht schon decket alle Müden,  und so still ist's um mich her,  Herr, auch mir gib endlich Frieden,  denn ich wünsch' und hoff' nichts mehr!  **[Herr, auch mir gib endlich Frieden,**  denn ich wünsch' und hoff' nichts mehr!] | Like a mortally wounded warrior,  who has lost his way,  I begin to falter and cannot go on,  Worn to death by Life.  Now, night blankets all my weariness,  And it is so silent around me;  Lord, grant me peace!  As I desire and hope for nothing more! |
| --- | --- |

## 5.3. Brahms: "O süßer Mai"

From "Sechs Lieder und Romanzen", Op. 93a Nr. 3

Composed: 1883

Voicing: SATB

Text: Achim von Arnim

Composer: Johannes Brahms

Recording / performer: Ensemberlino Vocale, conducted by Ulrich Kaiser

Recording duration: 1:57

| O süßer Mai,  Der Strom ist frei,  Ich steh verschlossen,  Mein Aug' verdrossen.  Ich seh nicht deine grüne Tracht,  Nicht deine buntgeblümte Pracht,  Nicht dein Himmelblau,  Zur Erd' ich schau.  O süßer Mai,  Mich lasse frei,  Wie den Gesang  An den dunkeln Hecken entlang. | O sweet May,  streaming freely,  I stand close-mouthed,  my eyes querulous.  I see neither your green array,  your colorfully blossoming splendour,  nor your blue skies;  to the earth I gaze.  O sweet May,  release me  like a song  along the dark hedge. |
| --- | --- |

# 6. Supplementary references

Kaiser, U. (2018). *Analyse des Chorliedes »O süßer Mai« Op. 93a von Johannes Brahms*. https://kaiser-ulrich.de/publicfiles/oer-texte/Brahms-Analyse-Op.93a-Nr.3.pdf

1. Department of Cognition, Emotion, and Methods in Psychology, University of Vienna, Austria
    * corresponding author: tudor.popescu@oxon.org
2. Department of Psychology, University of Graz, Austria
3. University of Music and Performing Arts, Vienna, Austria
4. Department of Psychology, University of Milan-Bicocca, Milan, Italy
5. Center for Mind/Brain Sciences (CIMeC), University of Trento, Rovereto, Italy
